# Helminth coinfection and COVID-19: An alternate hypothesis

**DOI:** 10.1371/journal.pntd.0008628

**Published:** 2020-08-17

**Authors:** Russell Hays, Doris Pierce, Paul Giacomin, Alex Loukas, Peter Bourke, Robyn McDermott

**Affiliations:** 1 Australian Institute of Tropical Health and Medicine, James Cook University, Smithfield, Australia; 2 Centre for Molecular Therapeutics, Centre for Tropical Bioinformatics and Molecular Biology, Australian Institute of Tropical Health and Medicine, James Cook University, McGregor Road, Smithfield, Australia; 3 Australian Institute of Tropical Health and Medicine, James Cook University, Smithfield, Australia; 4 Centre for Molecular Therapeutics, Australian Institute of Tropical Health and Medicine, James Cook University, Cairns, Australia; 5 Cairns Hospital, Cairns, Australia; 6 James Cook University, Cairns, Australia; 7 Division of Health Sciences, University of South Australia, Adelaide, Australia; New York Blood Center, UNITED STATES

In their recently published commentary, Bradbury and colleagues [1] drew attention to the possible negative interactions between helminth infection and COVID-19 severity in helminth-endemic regions. Helminth infections are known to be powerful modulators of the human immune response, and numerous studies now highlight the effects this may have on human infectious, inflammatory, and metabolic diseases. We believe, however, that any interaction between pre-existing helminth infection and the subsequent severity of COVID-19 need not necessarily be a negative one, and theoretical and empirical evidence suggests that helminths may indeed have a mitigating effect.

One of the clear predictors of severe COVID-19 that has emerged during the pandemic has been the presence of obesity, metabolic syndrome, or type 2 diabetes mellitus (T2DM) in patients contracting the virus. [2] These conditions are associated with the second week “cytokine storm” phenomenon [3] that often results in the need for ventilatory support and increased mortality, including in younger patients. These metabolic diseases are characterised by an inflammatory milieu, with increased levels of proinflammatory cytokines, many of which are also implicated in the severe form of COVID-19. Elevated levels of acute phase reactants and proinflammatory cytokines including IFN-ɣ, IL-1β, IL-6, IL-12, IL-17, IL-18, IL-27, and TNF have been shown to be predictors of clinical deterioration and the onset of severe disease. Lymphopaenia (and, in particular, a reduction in regulatory T cells [Treg] numbers) and eosinopaenia are also closely associated with disease severity. [4]

Epidemiological studies over the past decade have consistently reported an inverse relationship between a variety of chronic helminth infections and the presence of metabolic syndrome and T2DM, particularly in transitional societies in which both conditions are prevalent. [5] Recent reports have shown reduced levels of proinflammatory cytokines such as IL-1α, IL-1β, IL-6, IL-12, IL-18, IL-23, IL-27, G-CSF, and GM-CSF in subjects with coexisting helminth infection and T2DM and a partial reversal of this effect following treatment of the worm infection. [6] In addition, chronic helminth infections are associated with increased numbers of Treg cells, M2 macrophages, and eosinophils. It is therefore feasible to propose that a reduced capacity for the production of proinflammatory cytokines and increased numbers of regulatory immune cells due to the immunomodulatory effects of pre-existing helminth infection could result in a reduced risk of severe COVID-19.

Although the interaction between helminth infection and viral pneumonia is poorly defined, there is some evidence that helminth infection may moderate the process of pulmonary inflammation in viral infections. Some studies have suggested that helminth infection may impair responses to viral immunization [7] and viral infection [8], but no clear clinical evidence exists that it acts to worsen outcomes. [9] The increased levels of IL-4 and IL-10 (anti-inflammatory cytokines associated with chronic helminth infection) found in some studies [10] need not represent a virus induced pathological response but could instead reflect the normal regulatory and tissue-repair response to inflammation.

Epidemiological studies of the prevalence of severe COVID-19 in societies in which helminth infection is common would clearly be of great interest, but currently, no reliable data exists. The pandemic has been most active in developed countries where helminth infection is rare, and the data coming from less-developed societies may be difficult to interpret given the early phase of the pandemic, the lack of extensive testing, unreliable information regarding case fatality rates and cause of death, and their generally younger populations with lower prevalence of metabolic disease and obesity. The numbers currently emerging from the WHO do not indicate a widespread increase in case fatality rates in the developing world, with the number of reported deaths being generally low. [11]

We believe that, as the understanding of the mechanisms of severe COVID-19 evolves, there may be a case for exploring the possible effects of experimental helminth infection (EHI) on COVID-19 severity in a study setting. This is particularly so should the mechanism of severe disease rest principally in endothelial invasion and vascular injury secondary to unchecked inflammatory responses, rather than ongoing viral replication. [12] Although the outcomes of trials have been mixed [13], experimental inoculation with the hookworm *Necator americanus* has been established as both practical and safe for use in study settings and has been successfully deployed in trials involving atopic and autoimmune disorders. [14] Importantly, no data exists studying the effect on metabolic outcomes, but one study is currently underway examining the effect of EHI on individuals with obesity and metabolic syndrome. [15] In the present crisis, a prospective study examining the effect of EHI on subsequent severe COVID-19 could produce valuable insights into the immunology of this condition. Clearly, the design of such a study would pose considerable ethical and practical challenges. Experimental coronavirus infection would seem impossible, particularly given that the trial would logically target those at most risk of severe disease. A case-matched cohort study conducted at multiple locations around the world would require large numbers of subjects and would be dependent on the unpredictable future course of the pandemic. Nevertheless, such a trial could demonstrate a potential mitigation of severe disease in susceptible individuals and give some evidence-based guidance on how to best manage the helminth elimination programs currently operating in many countries as the pandemic unfolds over coming years.
